# A Scoping Review of Global Literature on Alcohol and Other Drug-Facilitated Sexual Violence

**DOI:** 10.1177/15248380241297349

**Published:** 2024-11-19

**Authors:** Jessica Ison, Ingrid Wilson, Kirsty Forsdike, Jacqui Theobald, Elena Wilson, Anne-Marie Laslett, Leesa Hooker

**Affiliations:** 1 La Trobe Rural Health School, La Trobe University, Melbourne, VIC, Australia; 2 Health and Social Sciences Cluster, Singapore Institute of Technology, Singapore, Singapore; 3 Center for Alcohol and Policy Research, La Trobe University, Melbourne, VIC, Australia; 4 Judith Lumley Center and La Trobe Rural Health School, La Trobe University, Melbourne, VIC, Australia

**Keywords:** sexual assault, alcohol and drugs, date rape, anything related to sexual assault, drug-facilitated sexual assault, women, drink spiking, trauma

## Abstract

The use of alcohol or other drugs to facilitate sexual violence (AOD-facilitated sexual violence) is a public health concern. There are significant gaps in knowledge on victimization, perpetration, contexts, impacts, and attitudes. Using a scoping review method, we mapped existing peer-reviewed, global literature to examine what is known about AOD-facilitated sexual violence to inform the development of AOD-facilitated sexual violence targeted interventions. We searched databases such as: Medline, PsycINFO, Scopus, ProQuest, PubMed, and EBSCO. Studies were included if they examined sexual violence where alcohol and/or other drugs were opportunistically or proactively used to facilitate sexual offending, within intimate partner and non-intimate partner (acquaintance or stranger) relationships. We reviewed 53 articles and mapped the findings to five knowledge areas: (1) prevalence; (2) victim-survivors and perpetrators; (3) location, nature, and substance used; (4) predictors, risk factors, and impacts; and (5) representations and attributions of blame. Although conclusions are difficult to draw due to the limited disparate literature, our review extends existing knowledge, highlighting that perpetrators are often known to victim-survivors, AOD-facilitated sexual violence also occurs in private locations, and alcohol is a common substance utilized in AOD-facilitated sexual violence (though its role is complex). Troubling myths and misconceptions about victim-survivors and negative representations in the media affect attributions of blame, particularly in cases where victim-survivors voluntarily consume substances. To inform interventions, this review identifies the need for consistent definitions and measurement of AOD-facilitated sexual violence, greater diversity of experiences, and the need to challenge attitudes that blame victim-survivors where substances are involved.

## Introduction

Alcohol and other drug-facilitated sexual violence (AOD-facilitated sexual violence) is a public health concern that can have lasting impacts on victim-survivors (Badour et al., 2020). There is inconsistent evidence on this nuanced form of sexual violence, which hinders efforts toward prevention (Hooker et al., 2021). This article reports on a scoping review of the global literature on AOD-facilitated sexual violence.

### Sexual Violence

Sexual violence is an umbrella term defined by the World Health Organization (WHO) as “any sexual act, attempt to obtain a sexual act, unwanted sexual comments or advances, or acts to traffic, or otherwise directed, against a person’s sexuality using coercion, by any person regardless of their relationship to the victim” (World Health Organization, 2013). Evidence of sexual violence broadly indicates that most perpetrators are male (Australian Institute of Health and Welfare, 2020), and victim-survivors are women, children, and LGBTIQ+ people. The WHO estimates that globally, 6% of women have experienced sexual violence perpetrated by a non-partner since the age of 15 years (World Health Organization, 2021). Although the WHO does not have global data on intimate partner sexual violence only, they do estimate that 26% of women have experienced physical and/or sexual violence from an intimate partner in their lifetime (World Health Organization, 2021). LGBTIQ+ sexual violence research is still emerging with little available global evidence, but country-specific data shows that some communities, such as bisexual women, experience rates similar to cisgender heterosexual women (e.g., Hill et al., 2020). Alongside this, people who experience multiple intersecting inequalities, such as racism or ableism, experience higher rates of sexual violence (Mitra-Kahn et al., 2016; Sutherland et al., 2021). However, it is well-recognized that prevalence estimates do not represent the full extent of sexual violence due to under-reporting (Lievore, 2003).

It is also difficult to report on prevalence rates of specific types of sexual violence due to underreporting and stigma. However, some research can offer insights into certain types of sexual violence, including perpetration. For example, a recent Australian study (Doherty & Christopher, 2024), with a sample of 5,076 respondents, asked about perpetration of sexual violence. It showed troublingly high rates of sexual coercion perpetration (18.2% men and 13.9% of women reported perpetrating “sexual harassment and coercion ever during adulthood” respectively with gender-diverse rates not reported). Male respondents also reported significantly higher rates of perpetrating nonconsensual kissing, touching, and stealthing (non-consensual condom removal), than female respondents.

### AOD and Sexual Violence

The co-occurrence of alcohol and sexual violence perpetration has been the subject of a considerable body of research (see reviews: Abbey, 2011; Lorenz & Ullman, 2016). Much of this research has focused on examining the role that *perpetrator* alcohol consumption and intoxication plays in sexual violence (Abbey et al., 2022; Parrott et al., 2023). This literature comprises studies of varying designs and reveals the complex factors and mechanisms in alcohol-involved sexual violence. Abbey et al.’s (2022) comprehensive review of this literature showed that the pharmacological effects of alcohol on cognitive functioning play a role in the interpretation of salient cues. Additionally, they showed that alcohol use, combined with perpetrator characteristics such as hostile masculinity and poor attitudes toward women, and sexual alcohol expectancies increase the likelihood of sexual violence. Furthermore, Abbey et al. (2022) showed that alcohol-affected perpetrators are more likely to use force and women are more likely to suffer injury and that incidents are more likely to occur in contexts where both perpetrator and victim are drinking (e.g., parties or bars). And there is a complexity of social norms around alcohol and casual sex, where alcohol is widely recognized as offering “liquid courage” to facilitate consensual sex (Abbey et al., 2022). It should be noted that much of this research is conducted using small samples, predominantly with North American heterosexual college students, and further research is needed to explore these issues with different populations (Parrott et al., 2023).

Research has also focused on the role of victim alcohol use as a risk factor for sexual violence where the physiological effects of alcohol increase women’s vulnerability to sexual violence in situations that involve motivated perpetrators (Lorenz & Ullman, 2016). Evidence shows differences in alcohol-involved assaults compared to non-alcohol involved assaults in decisions to disclose, responses by others and recovery from the assault, as detailed in Lorenz and Ullman’ (2016) comprehensive review. Lorenz and Ullman (2016) showed that women who have been drinking may be less likely to interpret or label the assault as sexual violence. In terms of disclosure, Lorenz and Ullman (2016) showed how impaired or intoxicated victims tend to disclose to informal sources rather than formal support sources. Also, sexual assault victims who have been voluntarily drinking commonly receive negative social reactions and may be blamed and disbelieved (Ullman & Filipas, 2001). Accordingly, the post-assault recovery by victims reveals self-blame, psychological distress, avoidant coping strategies, and subsequent problem alcohol consumption (Lorenz & Ullman, 2016).

### AOD-Facilitated Sexual Violence

There is increasing attention on the role of alcohol and other drugs to facilitate sexual violence. AOD-facilitated sexual violence refers to the use of alcohol or other drugs in the commission of an act of sexual violence. Colloquially it may be referred to as “drink spiking” and in the academic literature, it is often referred to as drug-facilitated sexual assault. In this article, we use the term AOD-facilitated sexual violence to encompass the following scenarios:

- Where substances may be administered to someone without their knowledge or consent, with the intent to incapacitate them and commit an act of sexual violence (these are often the common representations of “drink spiking”).
- When the victim may voluntarily drink alcohol or consume drugs and their ability to give free and informed consent to sexual activity may be compromised, which the perpetrator uses to facilitate sexual assault.
- Where the perpetrator plays a facilitative role in encouraging alcohol consumption, to the extent that the person is unable to consent to sex
- Where perpetrators act opportunistically (e.g., take advantage of a person who has voluntarily consumed alcohol and is already at least partially intoxicated) or proactively (e.g., premeditate their actions by adding a chemical known to cause incapacitation, so that it is ingested without the victim’s knowledge) (Gee et al., 2006).
- Where an individual is forced by others to sexually penetrate someone else (e.g., using a body part or object) when the victim is affected by substances and unable to consent (Basile et al., 2021).

We therefore draw a critical distinction here between the general field of AOD-related sexual violence to focus on where AOD is used to *facilitate* sexual violence. We use facilitate in the broadest sense while acknowledging that the terminology has limitations because sexual violence is such a complex phenomenon where perpetrator intentions may not be known. Thus, we use “facilitate” because it centers the actions of the perpetrator rather than the victim.

A further complicating factor is that the terminology to date has tended to use “drug-facilitated sexual assault” as the research has often been situated within studies of toxicology which can obscure the role of alcohol. Furthermore, popular narratives often referred to as “drink spiking” which indicates a stranger in a bar administering drugs to a victim. As we discuss below, alcohol is often used by the perpetrator to facilitate sexual violence. We want to draw attention to alcohol’s role while also shifting popular understandings away from drug-related drink spiking or other similar forms of perpetration. Therefore, we have chosen to put alcohol first in our terminology.

Lastly, there is considerable overlap in the literature between AOD-facilitated sexual violence and other forms of sexual violence such as incapacitated rape, which refers to when a victim is incapacitated from their own AOD use and they are sexually assaulted. Clearly, it is difficult to draw firm lines between AOD-facilitated sexual violence and incapacitated rape. All of this being said, we nonetheless note that terminology must be malleable and expansive to ensure the vastly different experiences of victim-survivors are accounted for.

There is a growing body of research on AOD-facilitated sexual violence. Most of the existing research focuses on examining the toxicological (Costa et al., 2020; Olszewski, 2009) and forensic legal aspects (Hall & Moore, 2008). A systematic review on “drug-facilitated sexual assault” drawing on quantitative studies using hospital administrative data, case file analyses, or toxicological reports found that the most common substance used is alcohol, the perpetrator is most likely a known male, and the victim is a female under 30 years of age (Recalde-Esnoz et al., 2023). A systematic review of the toxicology of drug-facilitated sexual assault (Anderson et al., 2017) found that alcohol was a common factor across the evidence. The use of alcohol includes a perpetrator proactively administering alcohol and opportunistically using a victim’s impairment to commit sexual violence (Abbey et al., 2022). Some of the other substances used to sedate victims include flunitrazepam (Rohypnol) or other benzodiazepines and gamma-hydroxybutyrate (GHB) (Wolitzky-Taylor et al., 2008). These central nervous system depressant drugs cause confusion, the inability to walk, amnesia, and unconsciousness. Not all instances of administering alcohol or other drugs result in sexual violence; however, it can interfere with the incapacitated person’s ability to consent to sexual activity.

Some research does suggest that AOD-facilitated sexual violence can occur in any setting, such as universities/colleges, private residences, parties, bars, and clubs (Anderson et al., 2017). However, media reports are often focused on bars and clubs, paired with reporting on stranger sexual assault, rather than the more likely scenario of the perpetrator being an acquaintance (Clinnick et al., 2024).

It is clear that AOD-facilitated sexual violence is under-reported, although much of the evidence is dated. For example, a 2011 United States of America (USA) national study of 3,001 shows the prevalence of reporting to be 1 in 10 (Wolitzky-Taylor et al., 2011). When a sexual assault does occur, there are considerable individual and structural barriers to victim-survivors reporting, across the health and criminal justice systems, such as not being believed or dismissed as having “drunk too much” (Fitzgerald et al., 2017; Weiss, 2010). Barriers to reporting relate to concerns around what constitutes “real rape”, victim blaming and not being believed, which are exacerbated when alcohol or other drugs are involved.

From a policy perspective, increasingly, governments are showing concern about the use of AOD to facilitate sexual violence (Donovan, 2016; House of Commons Home Affairs Committee, 2022; Loughnan, 2010). A recent national United Kingdom (UK) survey of both victim-survivors and witnesses to drink spiking (from 2016 to 2019) reported annual increases with 1,903 cases in 2019 (House of Commons Home Affairs Committee, 2022). Of note, however, the report focused on drink spiking, not associated sexual assaults.

AOD-facilitated sexual violence research has some clear gaps in knowledge on prevalence, victimization, perpetration, and risk factors, particularly when paired with negative media representations. Thus, there are challenges to designing appropriately targeted interventions and policy and system responses. Although there have been some targeted systematic reviews analyzing AOD-facilitated sexual violence, there are no studies that bring together the broader field of research, including a range of research designs. A scoping review offers the opportunity to widely survey the field using expansive search terms and inclusion criteria. As such, the primary aim of this study is to explore what is known internationally about AOD-facilitated sexual violence and to provide future research recommendations that can inform prevention and intervention efforts.

## Method

To adequately explore what is known globally about AOD-facilitated sexual violence, the authors undertook a scoping literature review. Scoping reviews are useful in identifying knowledge gaps, particularly on topics that have been studied across a wide range of fields, study designs, and contexts (Munn et al., 2018). This review followed the extended guidelines for a scoping review outlined by Levac et al. (2010), which build on Arksey and O’Malley’s (2005) six-stage framework. These steps included identifying the research questions, identifying the relevant studies, charting the data, collating and summarizing and analyzing the results. We report our results using PRISMA’s framework for reporting scoping reviews (Tricco et al., 2018).

The scoping review questions are:

1. What is known about AOD-facilitated sexual violence in the international literature?
2. What are the existing gaps in knowledge that can inform prevention and intervention efforts?

We opted for question one to be as broad as possible to allow for a scoping of the field. Question two was chosen to ensure the review was able to inform prevention and intervention efforts in the community. To identify relevant studies, we searched Medline, PsycINFO, Scopus, ProQuest, PubMed, and EBSCO bibliographic databases. The search date range was from 2005 to November 2023. The date was chosen as there is a gap in this period since one of the most significant national prevalence studies on drink spiking in Australia was published in 2004 (Taylor et al., 2004). Search terms were identified through firstly defining the two key constructs (alcohol or drug-facilitated and sexual violence) adapted from the Population, Exposure, Outcome framework (Moola et al., 2015). Second, we searched key constructs in databases to assess keywords. Third, we conducted a preliminary search of the existing literature and any terminology used in existing reviews (e.g., systematic reviews, scoping reviews). Fourth, the research team discussed and refined the terms, coming to a consensus. Lastly, the first and second authors conducted an initial search to explore further missed terms or phrases, and they compared the terms to ensure no search terms had been missed. The search terms are outlined in Table 1.

**Table 1.** Search Terms.

Key construct	Keywords
Alcohol or drug-facilitated	GHB OR “gamma hydroxybutyrate” OR flunitrazepam OR rohypnol OR ketamine OR benzodiazepine OR “drug facilitated” or drug-facilitated OR drugging OR “alcohol facilitated” OR alcohol-facilitated OR “drink spik*” OR drink-spik* OR DFSA OR “forensic toxicology” OR “covert drug administration” OR “needle spik*” OR “substance-facilitated” OR “substance facilitated”
Sexual violence	“Sex* violence” OR “sex* assault” OR “sex* abuse” OR “sex* harassment” OR rape OR “attempt* rape” OR “acquaintance rape” OR “date rape” OR “force* sex” OR “non-consen* sex” OR “sex* offense” OR “sex* crime” OR “sex* attack” OR “unlawful sex* conduct” OR “sex* coercion” OR “sex* consent” OR “sex* misconduct” OR “sex* exploitation” OR “indecent* assault*” OR “indecent* expos*” OR “revenge porn” OR sextortion OR “image based abuse” OR “image-based abuse” OR “dating violence”

A priori inclusion and exclusion criteria were determined as follows.

### Inclusion Criteria

Studies were included if they examined sexual violence (rape, sexual assault, sexual harassment, image-based sexual assault) where alcohol and/or other drugs were opportunistically or proactively used to facilitate the offense. This offense could occur within intimate partner and non-intimate partner (acquaintance or stranger) relationships. We included studies of any victim or perpetrator type (any age, sex/gender, race), any location of offence (e.g., home, licensed premises, festival, event etc.), and we did not limit studies by country. We included peer-reviewed primary research and theses (Doctorate and Masters) written in English.

### Exclusion Criteria

Studies were excluded if (a) they primarily focused on sexual violence with no involvement of alcohol and/or drugs; (b) the study did not consider the facilitative role of alcohol and/or drug as instrumental in sexual violence (e.g., alcohol and/or drug present but the victim was not incapacitated in the sexual violence/intimate partner violence/dating violence); and (c) intimate partner violence where it did not specify sexual violence. We excluded reviews and non-peer-reviewed material such as conference papers, commentaries and editorials, books, and websites.

### Study Selection

**Figure 1.**
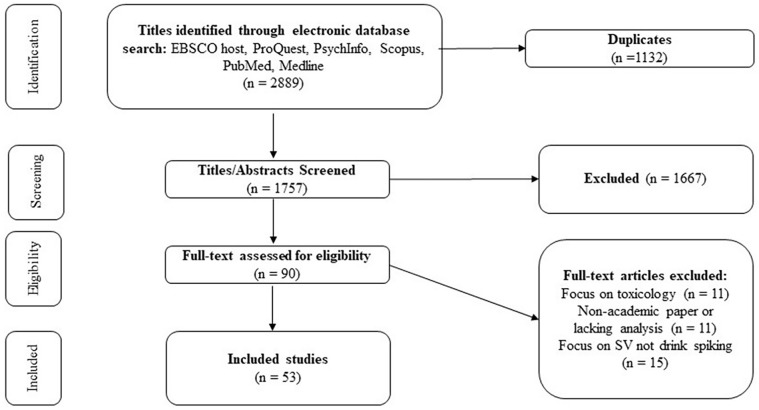
PRISMA flowchart of study selection.

As reflected in the PRISMA flowchart (Figure 1), 2,889 studies were downloaded into Covidence (Veritas Health Innovation, 2022). Duplicates were removed, and 1,757 studies remained. The first and second authors independently screened the titles and abstracts of the remaining studies and excluded 1,667 studies that did not meet the inclusion criteria. Full-text review of the remaining 90 studies was performed by the first and second authors. Discrepancies about inclusion of any studies were resolved by discussion of how inclusion and exclusion criteria were interpreted and applied, leading to consensus resulting in the exclusion of 29 articles. At this stage, we then opted to exclude a further eight quantitative studies that focused solely on toxicology results and chemical makeup of substances used, as these included little or no substantive discussion of the issue of AOD-facilitated sexual violence. We included toxicology case reports as these included information about the sexual violence victimization, whereas the toxicology-only articles were limited to analyzing the properties of the substances. We did not look at the reference lists to determine if we missed any studies. The final selection of studies included 53 studies for final extraction.

### Charting and Mapping the Studies

A data extraction table was created in Covidence, and the lead author extracted key elements from the studies, including aim, study design, sample and setting, and key findings. Studies were reviewed, and their findings organized into focus areas identified with consensus from the research team.

Before turning to the results, we offer a note on terminology. Throughout the research there is inconsistent terminology. Our preference is for AOD-facilitated sexual violence as discussed earlier. However, we have tended to use the terminology that each article uses to adhere to its meaning. As such, terminology differs throughout the results.

## Results

### Characteristics of Included Studies

The characteristics of the 53 selected studies are summarized in Appendix 1. The included studies were published between 2007 and 2023 with no relevant studies published in 2005 or 2006. Over half of the selected studies were conducted in the USA (*n* = 29, 54%). Five studies were conducted each in the UK and Canada and four in Spain. The remaining studies were conducted in Australia, Costa Rica, Denmark, India, Ireland, Italy, Nigeria, and South Africa. Studies came from a range of disciplines including psychology, criminology, forensic science, and gender studies.

The majority of included studies were quantitative (*n* = 42, 79%) of which 12 were descriptive analyses of sexual assault case records from toxicology reports (Anderson et al., 2019; Bidstrup et al., 2022; Caballero et al., 2017), law enforcement or judicial records (Cerdas et al., 2014; Quintana et al., 2020), or services such as health or sexual assault centers (Du Mont et al., 2009, 2016; Fields et al., 2022; Larsen & Hilden, 2016; Mognetti et al., 2022; Richer et al., 2017; Singh et al., 2014). One pilot study used a mixed methods approach exploring drug-facilitated sexual assault among university students (Burgess et al., 2009). Ten studies (19%) used qualitative approaches, including three studies that conducted media analyses of the construction of drug-facilitated sexual assault and date rape in British, USA, and Australian newspapers (Clinnick et al., 2024; Moore, 2009, 2011). Four quantitative studies (Crawford et al., 2008; Girard & Senn, 2008; Jenkins & Schuller, 2007; Stewart & Jacquin, 2010) and two qualitative studies (Angelone et al., 2007; Finch & Munro, 2007) explored responses to AOD-facilitated sexual violence scenarios in the context of mock jury trials or vignettes. Over one-third of studies featured university or college-residing student samples. Though studies may have included people 16 years and over in their cohort, only five articles were focused on young people under 18 years.

The type of AOD-facilitated sexual violence in the studies (Table 2) ranged from focusing only on sexual assault facilitated by drugs other than alcohol (*n* = 3), alcohol only (*n* = 2), and alcohol and other drugs (*n* = 47). One study (Moore, 2011) explored the meaning of date rape over time, noting that it included the involvement of drugs and alcohol.

**Table 2.** Type of Alcohol or Other Drugs Included in Studies.

Type of AODFSV	Studies
Drug-only facilitated sexual violence	Brooks, 2014; Fields et al, 2022; Prego-Meleiro et al., 2021; Swan et al., 2017
Alcohol-only facilitated sexual assault	Dumbili & Williams, 2020; Jaffe et al., 2022
Alcohol and other drug facilitated sexual violence	Anderson et al., 2019; Angelone et al., 2007; Badour et al., 2020; Basile et al., 2021; Bidstrup et al., 2022; Brooks, 2011; Burgess et al., 2009; Burke et al., 2023; Caballero et al., 2017; Cerdas et al., 2014; Champion et al., 2022; Clinnick et al., 2024; Cohn et al., 2013; Crawford et al., 2008; Du Mont et al., 2009, 2016; Finch & Munro, 2007; French & Neville, 2013; Gilbert et al., 2019; Gilmore et al., 2018; Girard & Senn, 2008; Gómez et al., 2022; Jenkins & Schuller, 2007; Krebs et al., 2009; Larsen & Hilden, 2016; Lawyer et al., 2010; Littleton et al., 2021; McCauley, Conoscenti, et al., 2009; McCauley et al., 2010; McCauley, Ruggiero, et al., 2009; Mgolozeli & Duma, 2019; Mognetti et al., 2022; Mokma et al., 2016; Moore, 2009; Quintana et al., 2020; Richer et al., 2017; Schramm et al., 2018; Singh et al., 2014; Stewart & Jacquin, 2010; Ullman et al., 2019; Walsh et al., 2013, 2016; Wolitzjy-Taylor et al., 2008; Zinzow, Resnick, Amstadter, et al., 2010; Zinzow, Resnick, McCauley, et al., 2010; Zinzow et al., 2012

AOD-facilitated sexual violence was defined and described in different ways across the studies. Forty-seven studies used terminology that emphasized the substance was used instrumentally as a tool, for example, alcohol or drug “facilitated” rape. Other terminology referred to the victim, such as “incapacitated” rape. In several studies, this terminology was used inter-changeably. The sexual violence terminology used was predominately “sexual assault” or “rape”. At times, articles would pair the terminology of drug-facilitated rape in tandem with other forms of sexual assault, for example, framing it as “drug-facilitated rape/incapacitated rape” and the findings would combine drug-facilitated rape and incapacitated rape. When incapacitated rape was differentiated, it referred specifically to a perpetrator sexually assaulting an incapacitated person who they had not administered alcohol or other drugs to. The remaining articles used “date rape,” “drink spiking,” “drugging,” or “ingestion of a chemical substance.”

The review of the 53 articles is presented under the following focus areas: Prevalence of AOD-facilitated sexual violence; Victim-survivors and perpetrators; Location, nature, and substance used; Predictors, risk factors, and impacts; Representations of AOD-facilitated sexual violence.

### Prevalence of AOD-Facilitated Sexual Violence

A small number of studies examined the prevalence of some form of AOD-facilitated sexual violence within nationally representative samples of adults (Basile et al., 2021; Gilmore et al., 2018; Zinzow et al., 2012; Zinzow, Resnick, Amstadter, et al., 2010) and youths (Wolitzky-Taylor et al., 2008). The most recent and comprehensive study conducted in the USA used victimization data from their 2010 to 2012 National Intimate Partner and Sexual Violence survey involving 41,174 adult respondents assessing lifetime and 12-month prevalence of stalking, sexual violence, and intimate partner violence. They found that, among victims of alcohol/drug-facilitated rape, 29.7% of adult females and 32.4% of male rape victims reported alcohol or drugs were used involuntarily in the first encounter with the perpetrator. For cisgender male victims made to penetrate another, 14.6% reported involuntary use of substances (Basile et al., 2021).

In their US national sample of 3,001 English- or Spanish-speaking women’s lifetime exposure to sexual violence, Zinzow, Resnick, Amstadter, et al. (2010) found that 15% reported history of forcible rape (*n* = 439), 3% a history of incapacitated rape (*n* = 91), and 2% a history of drug and alcohol-facilitated rape (*n* = 69). Though, in a later article from the same data, Zinzow et al. (2012) reported slightly different numbers with 10% for forcible rape.

Some studies used a combination of samples, for example, Gilmore and colleagues examined large national samples of college students (*n* = 2,000) and household-residing women’s (*n* = 3,001) lifetime experiences of sexual violence, finding that 14.5% of women in the total sample reported a lifetime history of forcible rape and 7% reported a lifetime history of drug- or alcohol-facilitated/incapacitated rape (Gilmore et al., 2018). Wolitzky-Taylor et al. (2008) found that from 3,614 youth (12–17 years) survey respondents, 1.6% had experienced dating violence in their lifetime; 0.1% of the sample reported experiencing drug-facilitated rape from a dating partner, with this figure higher for girls at 0.2%.

In the remaining studies, it is difficult to assess the extent of AOD-facilitated sexual violence as these mostly involved site-specific analyses of case records of sexual assault victims identified through law enforcement data or presenting at hospital-based emergency centers, rape crisis, or other medico-legal centers. Hence, the proportions of cases involving alcohol or other drug facilitation in the sexual assault varied greatly due to the heterogeneity of data collection and analysis.

### Victim-Survivors and Perpetration

#### Victim-Survivors and AOD-Facilitated Sexual Violence

Thirty-nine studies examined the experiences of AOD-facilitated sexual violence victim-survivors, and six studies examined both victimization and perpetration. The victim was overwhelmingly female across all types of studies, though there were limited studies focused on male victims of AOD-facilitated sexual violence. Only two studies (Larsen & Hilden, 2016; Mgolozeli & Duma, 2019) featured samples that were solely male sexual assault victims. No studies sought to understand non-binary or trans people’s experiences of AOD-facilitated sexual violence, though they may be included in the sample, generally with very low response rates (Burke et al., 2023).

Thirty-two of the 39 studies that reported on ethnicity had a majority white/Caucasian cohort. The majority of the cohorts were aged from 18 to 25 years. In terms of sexuality, LGBTIQ+ people were rarely investigated beyond collecting demographics. Only one study focused on the prevalence and risk of what they call drugging in “sexual minority” students within their larger cohort study (Schramm et al., 2018) and found that male “sexual minority” students were 72.9% more likely to report drugging victimization than heterosexual males.

Three qualitative articles gave women’s perspectives on victimization and the strategies they used to protect themselves and their awareness of AOD-facilitated sexual violence (Brooks, 2011, 2014; Gómez et al., 2022). This included watching their drinks at bars (Brooks, 2011, 2014) and the important role of friendship as a protective measure for prevention (Gómez et al., 2021).

#### Perpetrators and AOD-Facilitated Sexual Violence

No studies were found that solely focused on perpetrator accounts of AOD-facilitated sexual violence. Six studies asked respondents about their experiences of being a perpetrator and a victim of AOD-facilitated sexual violence. Perpetrators were overwhelmingly male with very low rates of self-identified, female perpetration (Badour et al., 2020; Dumbili & Williams, 2020; Girard & Senn, 2008; Prego-Meleiro et al., 2021; Swan et al., 2017). The highest rate of perpetration was reported by Badour et al. (2020), who found that of those who self-identified as perpetrators, 649 (8.5%) were male, contrasting with 386 (4.1%) females. It should be noted, however, that the survey question asked, “how often in the last 12 months have you had sexual activities with another high school student because she or he was drunk or on drugs?” which may account for the unexpectedly high rate of female perpetration, as the question appears open to broad interpretation. Across the studies on self-identified perpetration, four had a majority white/Caucasian cohort, and four were high school or university student cohorts.

Two studies that asked respondents about witnessing perpetration of AOD-facilitated sexual violence reported low perpetration rates (Gómez et al., 2022; Swan et al., 2017). For example, in a study of 6,064 college students (Swan et al., 2017), 1.4% of respondents identified that they or someone they knew put a drug in someone’s drink. Of these, 29.1% engaged in sexual activity with the victim, with a higher prevalence among male respondents.

Sixteen studies included victims’ reports about the person who perpetrated AOD-facilitated sexual violence. The perpetrator/s were overwhelmingly a known male (e.g., Anderson et al., 2019; Caballero et al., 2017; Champion et al., 2022; McCauley, Ruggiero, et al., 2009). Less commonly, they were an acquaintance or stranger (e.g., Basile et al., 2021).

Using alcohol or drugs to facilitate the victim’s incapacitation to perpetrate sexual violence was the most common tactic adopted in Badour et al.’s (2020) population-based study of 16,992 high school students. The awareness of alcohol as a tactic for incapacitation was also confirmed by male participants in qualitative interviews with 26 young people (Gómez et al., 2021), though they tended to talk about others using this tactic, rather than themselves.

### Location, Nature, and Substance Used

#### Location and AOD-Facilitated Sexual Violence

The location or setting where AOD-facilitated sexual violence took place was not universally captured across all the studies. In a study of 6,064 college students (Swan et al., 2017), the most common location reported was a house or apartment, followed by a fraternity (a male-only on-campus accommodation in US colleges), bar, dormitory, and sorority (a female-only on-campus accommodation in US colleges). Similarly, Anderson et al. (2019) in their examination of 204 clinical files and toxicological analysis of Australian sexual assault cases, found that 48% of AOD-facilitated sexual violence occurred in private residences compared to an outdoor location (13%), hotel or motel (7%), and public venues (5%), which was a similar finding in other studies (e.g., Champion et al., 2022; Mognetti et al., 2022). By contrast, in Caballero et al.’s (2017) analysis of 152 Spanish sexual assault case files, most AOD-facilitated sexual violence occurred in entertainment venues (42.3%).

#### Nature of Sexual Violence and AOD-Facilitated Sexual Violence

Twenty-two studies analyzed different categories of sexual assault including those involving AOD-facilitated sexual violence (Table 3). Common categories and variations other than AOD-facilitated sexual violence included incapacitated rape, which refers to rape where the victim is incapacitated due to consumption of alcohol and other drugs consensually taken by the victim, and forcible rape, which refers to rape where no alcohol or other drugs is involved. For those studies that compared these offenses, they generally aimed to assess differences between sexual violence offenses and associated impacts for the victim-survivors (e.g., Krebs et al., 2009; Lawyer et al., 2010; McCauley, Conoscenti, et al., 2009; McCauley et al., 2010).

**Table 3.** Categories of Sexual Violence by Study.

Categories	Study
- Incapacitated rape/drug alcohol-facilitated rape - Forcible rape	Cohn et al., 2013; Gilmore et al., 2018; Mokma et al., 2016; Walsh et al., 2013, 2016
- Incapacitated rape - Drug and alcohol-facilitated rape - Forcible rape	Badour et al., 2020; Champion et al., 2022; Lawyer et al., 2010; McCauley et al., 2010; McCauley, Ruggiero, et al., 2009; Zinzow, Resnick, Amstadter, et al., 2010; Zinzow, Resnick, McCauley, et al., 2010; Zinzow et al., 2012
- Incapacitated rape - Non-incapacitated	Burke et al., 2023; Gilbert et al., 2019
- Incapacitated sexual assault - Physically forced sexual assault	Krebs et al., 2009; Littleton et al., 2021; Mognetti et al., 2022
- Predatory drug-facilitated sexual assault - Non-predatory drug-facilitated sexual assault	Du Mont et al., 2016
- Alcohol/substance-facilitated sexual assault where victim and perpetrator are using AOD	Ullman et al., 2019
- Predatory drug-facilitated sexual assault - Non-predatory drug-facilitated sexual assault - Non-drug-facilitated sexual assault	Fields et al., 2022
- Sexual coercion (verbal, alcohol or drugs, physical)	French & Neville, 2013
- Drug-facilitated sexual assault - Forcible rape	Du Mont et al., 2009
Dating violence including: - Physical assault - Sexual assault - Drug and alcohol-facilitated sexual assault	Wolitzky-Taylor et al., 2008

One study found that more physical force, as evidenced by genital injuries, was used in AOD-facilitated sexual violence (Du Mont et al., 2009). By contrast, two studies found that less force was used in sexual assaults involving AOD (Gilbert et al., 2019; Richer et al., 2017) compared to other types of sexual assault and one study found no statistical difference (Fields et al., 2022). When comparing the types of physical abuse compared between those who had consumed only alcohol and those who had consumed alcohol and other drugs (whether voluntarily or involuntarily), Mognetti et al. (2022) found a higher risk for physical injury in those who had consumed alcohol.

When assessing whether a weapon was used in AOD-facilitated sexual violence, there was contrasting evidence. Du Mont et al. (2016) found limited examples of weapons present in an examination of AOD-facilitated sexual violence case files. Two studies (Du Mont et al., 2009; Richer et al., 2017) compared AOD-facilitated sexual violence to other rape tactics, and both found that AOD-facilitated sexual violence had lower rates of weapons present. Fields et al. (2022) found no statistical difference between weapons use in drug-facilitated sexual assaults and non-drug-facilitated sexual assaults.

The types of sexual assault were documented across nine studies. For example, in their analysis of hospital case files, Du Mont et al. (2009) compared sexual assault that was drug-facilitated with non-drug facilitated. They found the drug-facilitated cohort had higher rates of kissing and fondling (82.2%), cunnilingus or fellatio (50%) and oral, anal, or vaginal penetration (95.2%). In their study of undergraduate women, Walsh et al. (2013) found that for AOD-facilitated sexual violence, the assault type was most likely vaginal penetration, but the rates were lower than reported by Du Mont et al. (2009) at 46.5%.

Victim-survivor amnesia was explored in nine studies. For example, in their study of toxicology case files, Anderson et al. (2019) found that 40% of the 204 victims could not report on the type of assault they experienced due to amnesia. Most of the studies on amnesia were quantitative, providing limited further information on the impacts of amnesia. However, Ullman et al.’s (2019) study on 141 women’s experience of impairment has qualitative written descriptions of amnesia. In particular, participants talk about experiences of “blackouts” and how the perpetrator used their state of being unconscious or incapacitated to sexually assault them.

### Substances Used and AOD-Facilitated Sexual Violence

Data on the substances used in AOD-facilitated sexual violence came from a small number of studies that involved victim self-report and toxicology analysis from sexual assault case files (Anderson et al., 2019; Caballero et al., 2017; Mognetti et al., 2022) or self-reported data from AOD-facilitated sexual violence victims indicating voluntary and involuntary use of substances—where this occurred (Basile et al., 2021; Du Mont et al., 2009, 2016; Littleton et al., 2021; Ullman et al., 2019). The vast majority of studies included a more general question about whether the victim was incapacitated at the time, with no accompanying questions about the substances.

The few studies that looked closely at AOD at the time of the assault show that alcohol was most commonly consumed by victims prior to sexual assault. For example, Caballero et al. (2017) analyzed case records of females reporting sexual assault. Self-report and toxicology findings showed that ethanol was the most frequently identified substance (79%), followed by pharmaceuticals (47%) and illicit drugs (38%) with some cases involving a combination of substance types. With the high percentage of victims reporting voluntary consumption of ethanol, the authors suggest that sexual offending was most often opportunistic or unplanned. The voluntary usage of substances, primarily alcohol, was also found in Anderson et al.’s (2019) case file review. Toxicology analysis identified 14 (of 204) cases where substances were identified that the complainant did not report voluntarily consuming, suggesting covert administration of these drugs to facilitate sexual assault. Conversely, there was a higher rate of drugs other than alcohol in Mognetti et al.’s (2022) retrospective analysis of 222 case files from hospital presentations of substance intake related sexual assault. They found that while more than 80% of cases reported voluntary alcohol use, toxicology reports showed a higher percentage of other drugs present. They note that many of the victims came forward more than 24 hours later; thus, alcohol may have no longer be present.

Although most studies focused on toxicology or victim-reported substance use, Ullman et al.’s (2019) study aimed to assess the categories often used in studies of AOD-facilitated sexual violence. They found that the categories used in these studies were about whether the victim-survivor was: often unimpaired (perceived no effect of drinking), impaired (conscious, but impacted by substance use), or incapacitated (unconscious due to substance use). Interviews with 141 women who were drinking and/or using substances at the time of the assault revealed that victims’ stories did not always align with these categories, often talking about being both impaired and incapacitated, or describing any of these categories.

Some studies had a general question about whether the victim recalled if the perpetrator or perpetrators were also intoxicated. An in-depth study from Littleton et al. (2021) of 344 undergraduate women who were victim-survivors, developed a typology of analyzing the substance use of both victims and perpetrators. This typology includes both force and voluntary substance use to highlight the interplay of victim and perpetrator substance use.

There was limited exploration of perpetrator motivations and methods of AOD-facilitated sexual violence. However, the motivations and practice of drugging were explored in one study that used a large sample of university student (*n* = 6,624) (Swan et al., 2017). A small number (1.4%) reported drugging someone (or being aware of another perpetrator drugging). They reported a mix of licit and illicit drugs used in AOD-facilitated sexual violence, including, for example, Rohypnol, Xanax, ecstasy, cocaine, Adderall, laxatives, and alcohol (Swan et al., 2017). The mode of drugging was also explored by Du Mont et al. (2016) who analyzed victim reports from hospital-based sexual assault treatment centers. They found that survivors of predatory drug-facilitated sexual assault were more likely to suspect they had been drugged via a recreational drug/non-alcoholic drink rather than an alcoholic drink.

Although survey questions may use terminology that included alcohol, the role of alcohol as a tool in facilitating AOD-facilitated sexual violence was rarely identified by people, though some qualitative studies did offer unique insights. When alcohol was discussed, it was clear that perpetrators had a clear understanding of how they could use alcohol to facilitate sexual violence. For example, offering women alcohol with the intent to facilitate date rape was disclosed by nearly all the 22 male participants in a study of 31 university students (Dumbili & Williams, 2020). Similarly, a study of 2,355 youths found men were more willing to have intercourse with someone who could not consent due to drugs and saw women’s use of alcohol and drugs as the cause of/justification for sexual violence against them (Prego-Meleiro et al., 2021).

### Predictors, Risk Factors, and Impacts

#### Predictors and Risk Factors

Five studies examined predictors of AOD-facilitated sexual violence which included past exposure to sexual assault. This included childhood abuse, adolescent abuse, or sexual assault before entering college. There is contrasting evidence on the rates of child sexual abuse experienced by those who have experienced different types of sexual violence. For example, Gilbert et al. (2019) compared incapacitated rape to non-incapacitated rape and found that (from a sample of 253 college women), non-incapacitated rape victim-survivors had higher rates of sexual abuse in childhood and before entering college. In contrast, Walsh et al. (2013) found in a larger sample (*n* = 714) of college women (those who had experienced child or adolescent sexual abuse [*n* = 226]), were more likely to report alcohol and other drug-facilitated rape, compared to those who had experienced forcible rape only (non-drug-facilitated). These findings were similar to Mokma et al. (2016). To further complicate these contrasting findings, Fields et al. (2022) found no statistically significant difference in rates of child sexual abuse between drug-facilitated sexual assault and non-drug-facilitated sexual assault victims, from a case file sample of 74 adults.

A few studies explored the risk factors contributing to AOD-facilitated sexual violence. For example, Du Mont et al. (2009) identified sexual assault cases involving suspected intentional drugging from cases presenting to hospital-based sexual assault treatment centers. They found that compared with other victims, victims of drug-facilitated sexual assault were more likely to present to large urban treatment centers, to have consumed over-the-counter medications and street drugs in the last 3 days before examination, and to have used alcohol before the assault. In a subsequent study, Du Mont et al. (2016) found that the odds of having experienced predatory drug-facilitated sexual assault were higher if the survivor self-reported mental health problems in the previous 6 months or that the mode of suspected drugging was a recreational drug or non-alcoholic drink (vs. an alcoholic drink).

Mental health has been identified as a risk factor for experiencing AOD-facilitated sexual violence. In their analysis of sexual assault victim toxicological files, Anderson et al. (2019) found that half of the complainants of drug-facilitated sexual assault (*n* = 204) had pre-existing mental health conditions. Mental health, especially hyperarousal as part of post-traumatic stress disorder (PTSD), has also been associated with victim-survivors of AOD-facilitated sexual violence. Walsh et al. (2013) analyzed the impact of childhood or adolescent sexual abuse on PTSD symptoms in a sample of 714 female university students, finding that PTSD is a strong mediating factor between a history of childhood or adolescent sexual assault and drug and alcohol-facilitated rape/incapacitated rape.

Although the research often focused on issues such as childhood sexual abuse or mental health as a risk factor, there was also some engagement with people’s behavior at the time of the assault or their general behavior when they are in a public venue. In terms of the victim’s behavior before the assault, Gilbert et al. (2019) looked at risk factors for both incapacitated and non-incapacitated sexual assaults among university women. They found that incapacitated sexual assault victim-survivors, compared to those non-incapacitated, were more likely to have been at a party before the assault, and that the perpetrator was an acquaintance or friend, who was more likely to be using alcohol or other drugs.

The analysis of people’s general behavior, not necessarily just the behavior of those who had been victimized, extended to their general drinking behavior. One study looked at links between college student pre-partying behavior—drinking before an event–and incapacitated rape (Jaffe et al., 2022). Pre-party drinking was found to be a prospective risk factor for incapacitated rape, consistent with literature showing an association between drinking and risk for subsequent sexual assault.

People’s behavior in public also extended to the perception of risk, particularly from women, and the precautionary measures they may take to feel safe. The most common precautionary measure participants reported was monitoring their drinks (Brooks, 2011, 2014; Burgess et al., 2009; Crawford et al., 2008).

#### Impacts of AOD-Facilitated Sexual Violence

Seven studies examined the impact of AOD-facilitated sexual violence on victim-survivors, exploring associations with substance use, including alcohol consumption. A consistent finding across these studies was that an increase in problematic substance use was associated with AOD-facilitated sexual violence victimization. Having a history of incapacitated rape has been associated with more alcohol consumption and blackouts when pre-partying, as reported in Jaffe et al.’s (2022) study of 1,074 college students. There was some evidence that victims of drug and alcohol-facilitated sexual violence and incapacitated rape were associated with higher rates of current alcohol problems such as binge drinking, particularly for females, in Badour et al.’s (2020) population-based study of 16,992 high school students.

The impact of AOD-facilitated sexual violence on victim’s mental health was also examined. Seven studies found associations between AOD-facilitated sexual violence victimization and poor mental health outcomes in female victims, particularly PTSD, depression, and suicidal ideation. For example, from a national sample of 3,001 women, both drug and alcohol-facilitated rape and incapacitated rape have been associated with an increase likelihood of PTSD (Zinzow et al., 2012; Zinzow, Resnick, McCauley, et al., 2010). Another study compared the difference between Black and White respondents, finding that Black students (*n* = 107) who were victims of substance-coerced sexual assault had lower self-esteem and higher psychological distress than White students (*n* = 114) with respondents from both university and high school (French & Neville, 2013).

Victim-survivor help-seeking behavior was explored across three studies (Cohn et al., 2013; Richer et al., 2017; Walsh et al., 2016). Women who experience drug or alcohol-facilitated rape or incapacitated rape were less likely to acknowledge it as rape, consequently affecting whether to report to police (Cohn et al., 2013) or seek support from services (Walsh et al., 2016). Walsh et al. found that only one-third of female victims of rape (*n* = 445) sought help from services, but those who experienced drug and alcohol-facilitated rape/incapacitated rape were even less likely than those who experienced forcible rape to seek help. By contrast, Richer et al. (2017) analyzed 390 patients suspected of drug-facilitated rape who presented to a rape center over a 2-year period. They found that drug-facilitated sexual assault victims presented sooner, and more often attended medical follow-up and psychotherapy, than non-drug-facilitated sexual assault victims.

### Representations of AOD-Facilitated Sexual Violence and Blame Attributions

Several studies examined broader representations of AOD-facilitated sexual violence in the media and societal attitudes toward these offences and their victims, and implications for the attribution of blame in criminal justice system processes.

Media representations of drug-facilitated sexual assault and drink spiking were the subject of three studies. Of these, Moore (2009, 2011) analyzed the representation of drug-facilitated sexual assault in British and US media articles finding increasing representation of drug-facilitated sexual assault in the media over time. These representations focused mostly on the (female) victim and how she should have behaved, often drawing on individual narratives to act as a “cautionary tale” to other women. The author argues that this media attention contributes to the belief of a heightened threat of drug-facilitated sexual assault that is perhaps not aligned with the statistical reality of being a victim. A more recent study (Clinnick et al., 2024) analyzed the media reporting on AOD-facilitated sexual violence in Australian news media over a 10-year period (2012–2022). The study showed that the “cautionary tale” persists, as does victim-blaming and that victims reported a lack of care or support from emergency services.

Studies on societal attitudes discussed AOD-facilitated sexual violence and implications for attributions of blame or culpability in criminal justice settings. Varied methods were used to capture attitudes, including vignettes (Angelone et al., 2007; Crawford et al., 2008; Girard & Senn, 2008; Jenkins & Schuller, 2007), mock juror trials (Finch & Munro, 2007; Stewart & Jacquin, 2010), or questionnaires (Moore, 2009; Prego-Meleiro et al., 2021). Overall, these studies found that people had negative attitudes toward women who ingested substances voluntarily before they were sexually assaulted. Some of these negative attitudes extended to attributing blame for sexual violence to women’s use of alcohol or other drugs (Prego-Meleiro et al., 2021).

Other studies demonstrated the implications of these prevalent community attitudes when individuals seek justice. In a study where participants took part in a mock rape trial as the jury, they were given six scenarios involving the complainant consensually or not consensually ingesting a “chemical substance,” to determine if this impacted their decisions (Stewart & Jacquin, 2010). Across the six versions of the mock rape trial, participants viewed the complainant as less credible if she willingly used alcohol or drugs. However, if she non-consensually ingested a substance, she had higher credibility (particularly if this involved GHB and marijuana) but was still viewed as holding some culpability for the sexual assault. Those participants who endorsed rape myths tended to also rate the defendant (perpetrator) as more credible. These findings were consistent with Finch and Munro (2007), who also found that perpetrator intent increased jurors’ negative impressions of the perpetrator (e.g., the use of Rohypnol was viewed as holding clear malicious intent and was seen as premeditated).

## Discussion

This article reviews evidence of what is known internationally about AOD-facilitated sexual violence to inform future prevention and intervention efforts (see Table 4 for overview of critical findings). It is clear from this review that research on AOD-facilitated sexual violence is piecemeal; therefore, we can only draw limited conclusions. The overwhelming majority of studies were conducted in the USA using samples of college students. These samples comprised predominantly white, cis-gender heterosexual persons under 25 years of age. Only one study included the experience of “sexual minority” students. Hence, less is known about the experience of AOD-facilitated sexual violence from diverse groups, despite evidence of the intersectional nature of sexual assault risk and impact (Kammer-Kerwick et al., 2019; Sutherland et al., 2021; Ussher et al., 2020).

**Table 4.** Critical Findings.

- The field of AOD-facilitated sexual violence is disparate with studies overly focused on victims, in the context of US college students with a lack of diverse cohorts. - Rates of AOD-facilitated sexual violence were found to be higher among women, though limited studies of LGBTIQ+ communities exist. - Most perpetrators are male, and there was awareness by men of the tactic of using alcohol to facilitate sexual violence. - AOD-facilitated sexual violence occurs in the context of both voluntary and involuntary substance use. - Media reporting on AOD-facilitated sexual violence perpetuates a troubling focus on victim behavior. - Research examining predictive and risk factors had mixed findings. - The impact of AOD-facilitated sexual violence includes mental health outcomes such as PTSD and suicidal ideation.

The reported prevalence rates varied amongst the few nationally representative studies and should be interpreted with caution. The significant heterogeneity across studies means the real prevalence of AOD-facilitated sexual violence remains unclear. Furthermore, any reported prevalence rates are likely to significantly under-represent the true extent of AOD-facilitated sexual violence, which is reflected in broader sexual violence prevalence data (Orchowski et al., 2022). Studies did not have consistent definitions or measures of AOD-facilitated sexual violence and often relied on self-reported data from victims. A challenge in measuring prevalence is that AOD-facilitated sexual violence involves ingestion of incapacitating substances (whether alcohol or other drugs), which can affect short-term memory loss (Anderson et al., 2017), influencing victims’ reporting behavior.

Our review shows that the evidence has mixed results around help-seeking behavior with one study finding victims of AOD-facilitated sexual violence were less likely to seek help, whereas another found the victims of AOD-facilitated sexual violence presented to sexual assault services sooner. Research on help-seeking for all forms of sexual violence shows that victims face considerable barriers, particularly due to self-blame (Zinzow et al., 2022). There was also conflicting evidence on risk and predictive factors, for example, there were conflicting results on the impact of child sexual abuse on AOD-facilitated sexual violence. One concern with the focus on risk and predictive factors is that research can, however unintentionally, infer victim-blaming due to focusing on what a victim did, or did not do, before, during, and after an assault.

Despite mixed results, some consistencies were reported across the studies. The victims of AOD-facilitated sexual violence were mainly female (though we only found a small number of studies focusing on male victims and no studies on trans and non-binary people) and the perpetrators were male, likely known to the victim. AOD-facilitated sexual violence does not only occur in entertainment venues but in private residences (e.g., parties) and on-campus accommodation (college students). This contrasts with the common narrative in the media and popular discourse of the predatory stranger covertly administering an incapacitating drug within an entertainment setting with the intention to sexually assault an unsuspecting female (Clinnick et al., 2024). Our review revealed a more complex and nuanced picture of AOD-facilitated sexual violence compared to media representations—studies showed that AOD-facilitated sexual violence occurs in the context of voluntary *and/or* involuntary use of substances by the victim, and sexual offending may be planned or opportunistic where the victim is incapacitated to the extent of not being able to give consent. Although licit and illicit drugs are found to be used in predatory AOD-facilitated sexual violence (e.g., drink spiking), alcohol is a common substance involved in AOD-facilitated sexual violence (Abbey et al., 2022) and is most often voluntarily consumed by victims. Importantly, our review includes articles that found examples of men encouraging women to consume excess alcohol to facilitate sexual predation. This suggests the need to focus attention on the culture of men’s drinking and sexual entitlement, and to explicitly broaden awareness strategies to highlight alcohol as a tool for sexual violence.

This article extends other reviews of AOD-facilitated sexual violence (e.g., Recalde-Esnoz et al., 2023) by also including research that focuses on how AOD-facilitated sexual violence is represented in public discourse and the implications for criminal justice responses (particularly juries), which to date has often been overlooked. These studies revealed how media representation of AOD-facilitated sexual violence has grown over time, yet the focus remains on the victim’s actions and behaviors rather than that of the perpetrator (Easteal et al., 2014; Kitzinger, 2004). This emphasis is somewhat mirrored by our review which found *no studies* solely aimed at understanding perpetrator behaviors, which reflects the difficulty conducting research on perpetrators. This focus in public discourse has broader implications for potential handling of cases of AOD-facilitated sexual violence within justice settings. The studies of mock juror responses revealed a willingness to attribute blame to the perpetrator in cases that clearly met the accepted narrative of AOD-facilitated sexual violence noted above, whereas negative attitudes prevailed toward women who were assaulted after voluntarily ingested alcohol or other drugs. These findings feed into entrenched rape myths (Brownmiller, 1976) that blame victims and excuse men’s behavior; research shows that men endorse rape myths and victim blame more than women (Australian Bureau of Statistics, 2017). Women who eschew traditional gender roles and who consume alcohol before a sexual assault are attributed higher levels of blame (Grubb & Turner, 2012).

There were mixed findings about the use of physical force (as evidenced by the severity of physical injury) and weapons in AOD-facilitated sexual violence compared to other forms of sexual assault. Further investigation is needed in relation to perpetrators’ motivations and actions and the extent to which victim’s incapacitation was a factor in the nature of the offending behavior. In relation to the impact of AOD-facilitated sexual violence, it was associated with serious mental health outcomes, particularly PTSD, depression and suicidal ideation, and problematic alcohol use as a form of coping, consistent with other forms of violence and sexual assault (Oram et al., 2017). Further research—particularly qualitative research—could expand on this outcome-focused research by exploring victim-survivor resilience, specifically in the context of AOD-facilitated sexual violence.

### Future Research to Inform Prevention and Intervention

Our review identified several gaps for further research to inform prevention strategies and interventions (see Table 5 for an overview of implications for practice, policy, and research). Interestingly, our review did not identify any peer-reviewed research or evaluation studies of interventions designed to tackle AOD-facilitated sexual violence, despite examples such as drink spiking being identified as an issue of increasing public concern. We signal this as an area of future research and advocacy.

There is a need for more consistent definitions and corresponding measurement tools to better determine the prevalence of AOD-facilitated sexual violence (Recalde-Esnoz et al., 2023). Studies of prevalence should move beyond college samples to include children, adolescent, young, and older adults and specifically seek more diverse samples to better understand the breadth, extent and intersectional experience of AOD-facilitated sexual violence by minority populations. The extent to which AOD-facilitated sexual violence occurs within intimate partner relationships is a further area to explore in line with the emerging focus on sexual violence and intimate partner violence (Tarzia, 2021). For example, a recent case in Singapore involving the drugging and sexual assault of female spouses highlights the existence of such offending (Lum, 2023).

Quantitative studies have dominated the research landscape; in-depth qualitative research is needed that draws perspectives of victims representing diversity in relation to factors such as age, sexual orientation, ability, ethnicity, and class to understand their experience of AOD-facilitated sexual violence, impact, help-seeking, and support needs. The views of key stakeholders such as those providing services to victims would also provide important insights to inform community responses or evidence-based interventions.

**Table 5.** Implications for Practice, Policy, and Research.

- Future research would benefit from consistent definitions - Improved measurement is needed to accurately understand the true prevalence - Future studies must include an intersectional focus to better understand the breadth of the issue - Targeted prevention and response interventions must be developed which are tested and evaluated - Future studies should include in-depth qualitative research to broaden our understanding of AOD-facilitated sexual violence - Little is known about perpetration of AOD-facilitated sexual violence. Future research must include a focus on perpetration, including the role of harmful alcohol cultures - Advocacy is needed to address the media misrepresentation and troubling narratives of AOD-facilitated sexual violence

Finally, the review highlighted the significant work needed to change the narrative around AOD-facilitated sexual violence which garners sensationalized media attention intermittently (often focusing on drink spiking) (Our Watch, 2019). There is a need to address the culture and attitudes that foster men’s endorsement of rape myths, and where alcohol is viewed as a well-accepted tool to facilitate women’s sexual availability, to prevent AOD-facilitated sexual violence (Hooker et al., 2020). Societal attitudes that see women as culpable in their victimization because they have eschewed traditional gender roles by voluntarily using substances, must be changed in order for victim-blaming narratives to be disrupted and for women to experience justice.

## Strengths and Limitations

This review comprises a comprehensive and systematic assessment of existing global evidence, utilizing established scoping review methods. Other strengths include using a broad exploration of the research, a comprehensive search strategy and the inclusion of diverse study designs. The review has several limitations. First, only academic peer-reviewed studies published in English were included, potentially missing important grey literature and insights on AOD-facilitated sexual violence from low- and middle-income countries and cultural nuances. Furthermore, as with any review, there is a possibility that the search strategy could have failed to capture some research. Second, we excluded studies that focused on the toxicology of substances detected through forensic analysis, which represented a large proportion of the existing research as they rarely included any analysis on the sexual violence.

## Conclusion

The use of alcohol or other drugs to reduce someone’s capacity to consent to sex and facilitate sexual violence is a fundamental breach of human rights and individual sexual autonomy. There is growing attention to AOD-facilitated sexual violence globally, and this review examined existing literature from 2005 to 2023 to inform the development of prevention and intervention strategies. Despite the disparate literature, our review highlights that offenders are likely to be a known male, and sexual violence may take place in private settings and entertainment venues. In addition, alcohol is likely the most common substance involved in AOD-facilitated sexual violence, and it is a recognized tool within male drinking cultures for facilitating sexual predation. For victims to receive justice, there is a need to disrupt narratives and representations of AOD-facilitated sexual violence that sensationalize and blame victims, particularly where they voluntarily consume substances. Future research should establish the prevalence of AOD-facilitated sexual violence using consistent definitions and measurement tools. Ensuring the experiences of diverse groups are included in both quantitative and qualitative research on this topic is critical to developing tailored interventions.

## Supplemental Material

sj-docx-1-tva-10.1177_15248380241297349 – Supplemental material for A Scoping Review of Global Literature on Alcohol and Other Drug-Facilitated Sexual Violence Supplemental material, sj-docx-1-tva-10.1177_15248380241297349 for A Scoping Review of Global Literature on Alcohol and Other Drug-Facilitated Sexual Violence by Jessica Ison, Ingrid Wilson, Kirsty Forsdike, Jacqui Theobald, Elena Wilson, Anne-Marie Laslett and Leesa Hooker in Trauma, Violence, & Abuse
