# Supplementary material for: A Scoping Review of Global Literature on Alcohol and Other Drug-Facilitated Sexual Violence
Source: Trauma Violence Abuse. 2024 Nov 19;26(5):889–906. doi: 10.1177/15248380241297349 (PMC12569121; doi:10.1177/15248380241297349)
Supplement: sj-docx-1-tva-10.1177_15248380241297349 – Supplemental material for A Scoping Review of Global Literature on Alcohol and Other Drug-Facilitated Sexual Violence [file sj-docx-1-tva-10.1177_15248380241297349.docx]

## **Appendix 1** Summary of studies included in the review

| **Author, year** | **Country** | **Study aim** | **Study design & methods** | **Sample and setting** | **Key Findings regarding AODFSV** | **Type of AODFSV** |
| --- | --- | --- | --- | --- | --- | --- |
| Anderson, L.J., Flynn, A., Drummer, O., Gerostamoulos, D. & Schuman, J.L. (2019) | Australia | To examine complainant-specific and contextual factors and toxicological profile of drug-facilitated sexual assault (DFSA) in Victoria | Quantitative cross-sectional descriptive analysis of case file review | Victorian Institute of Forensic Medicine toxicology reports of 204 adult complainants (18 years and older) of alleged DFSA who underwent forensic examination between 2011 – 2013. | Complainants were female (93%); assailants were male. Most complainants had voluntarily consumed substances before DFSA. Alcohol was most common substance. 14 cases identified in toxicology tests suggest covert drug administration. | Drug-facilitated sexual assault (DFSA) |
| Angelone, D. J., Mitchell, D. & Pilafova, A. (2007) | USA | To examine, through vignettes, whether voluntary or involuntary ingesting alcohol or GHB effected participant perspectives on victimization and perpetration of sexual violence. | Quantitative analysis of responses to scenarios | 198 undergraduate students (18-48 years old). | In general, they perceived the scenario as rape and the perpetrator as responsible, regardless of voluntary or involuntary ingestion or the substance. Though there was a significant difference by gender, with females attributing less culpability to the victim. | Club drug use |
| Badour, C.L., Bell, S.C., Clear, E.R., Bush, H.M., & Coker, A.L. (2020) | USA | To investigate sex differences in associations between sexual violence victimization (SVV), sexual violence perpetration (SVP), and binge drinking and/or alcohol problems among high school students. | Quantitative cross-sectional analysis of 2011 data | 16,992 high school students from 26 high schools in Kentucky | Male SVP was 10.8% versus SVP by females (5.2%); female SVV was 21.2% versus male SVV (13.3%). More females experienced SVV involving alcohol/drug facilitated or incapacitated sex (9.3%) compared to males 8.2%; whereas SVP using these tactics was higher in males 8.5% compared to females 4.1% | Alcohol and drug-facilitated rape (ADFR) |
| Basile, K.C., Smith, S.G., Liu, Y., Lowe, A., Gilmore, A.K., Khatiwada, S. & Kresnow, M. (2021) | USA | To examine lifetime and 12-month prevalence the rape of women and men, and men who have been made to sexually penetrate someone, where alcohol or drug use is involved | Quantitative | 41,174 respondents from the 2010-2012 National Intimate Partner and Sexual Violence survey | Among victims of alcohol/drug-facilitated rape, 29.7% of adult female rape victims and 32.4% of male rape victims reported that alcohol or drugs were used involuntarily in at least one first encounter with the perpetrator. For male victims of being made to penetrate another, 14.6% reported involuntary use of substances. | ADFR |
| Bidstrup, J. E., Busch, J. R., Munkholm, J. & Banner, J. (2022) | Denmark | To explore whether closure of night venues during lockdowns caused a decrease in number of clinical forensic examinations of victims of SA and whether there was a change in characteristics of cases | Case file analysis of clinical forensic examinations | 130 cases of adolescent (>15 years) and adult complainants of sexual assault (1 April to 30 June 2019 and 2020). | Ratio of possible DFSA was approximately 50% of sexual assault cases forensically examined each year. No decrease in possible DFSA-cases after lockdown | DFSA |
| Brooks, O. (2014) | UK | To understand the views, experiences and behaviors of young women in relation to their safety while socializing in bars and clubs in Scotland | Qualitative research, focus groups and interviews | 35 women (18-25 years) | Women’s behaviors were complex and contradictory. Themes: Common sense and necessity; the inevitability of male behavior, disparity between theory and practice; maintaining control and respectability; resentment of focus on women’s behavior | Drink spiking (DS) |
| Brooks, O. (2011) | UK | To explore the views, experiences and behaviors of young women in relation to their safety when socializing in bars, pubs and clubs | Qualitative research, focus groups and interviews | 35 women (18-25 years) | Women endorsed and advocated safety behaviors identified within the prevention literature, however their behavior was complex and contradictory. | DS |
| Burgess, A., Donovan, P. & Moore, S. E. H. (2009) | UK | Pilot study to explore the nature, extent and basis of university student concerns about DFSA. | Quantitative descriptive and qualitative study using questionnaires interviews and focus groups | 236 undergraduate students from three UK universities | 10 of 236 experienced drink spiking with no sexual assault; high levels of awareness and risk. Active steps taken to avoid drink spiking, particularly by females. | DFSA |
| Burke, L. Dawson, K., Flack, W. F., O’Higgins, S., McIvor, C. & MacNeela, P. (2023) | Ireland | To assess whether substances use behaviors increase the risk of sexual violence and if so which substances are the most risky. | Quantitative cross-sectional sexual experiences survey | 1778 first year college students who participated in intervention | High rates of sexual violence for women and non-binary university students with incapacitations as the mostly frequently reported tactic. | AODFSV |
| Caballero, C. G., Jorge, Ã. Q. & Landeira, A. C. (2017) | Spain | To analyze the cases of DFSA at the National Institute of Toxicology and Forensic Sciences in Madrid between 2010-2013 | Retrospective case file analysis | 152 alleged sexual assault cases between 2010 and 2013 | Identified a common victim profile – young women drinking alcohol in entertainment venue prior to sexual assault with total or partial amnesia | DFSA |
| Cerdas, L., Arroyo, C., Gómez, A., Holst, I., Angulo, Y.,   Vargas, M., Espinoza, M. & León, G. (2014) | Costa Rica | To characterize victims, perpetrators and circumstances surrounding the cases of rape that occurred in Costa Rica | Prospective case report analysis | Randomly collected 272 reports of rape received by Judicial Investigation Agency between July 2012 and June 2013 | 259 records contained information about the involvement of drug and alcohol. 14% of rapes classified as “proactive drug-facilitated rape” and 25% classified as “opportunistic drug facilitated rape” | Drug-facilitated rate (DFR) |
| Champion, A., Oswald, F. & Pedersen, C. L. (2022) | Canada | To determine the frequency of sexual assault among Canadian female university students. | Quantitative | Nonprobabilistic, self-selected sample of 377 university women | Report a high number of sexual assaults, with the most commonly reported incidents being incapacitated sexual assault (where victim voluntarily consumed AOD). Sexual assault victimization highest among sexual minority women. | Substance-related sexual assault |
| Clinnick, I., Ison, J. &Hooker L. (2023) | Australia | To investigate the portrayal of drink spiking in Australian news media. | Media analysis. Qualitative content analysis | Australian newspapers over a 10-year period | The media perpetuates troubling narratives about AODFSV that include victim-blaming, racism and other forms of discrimination. Victim reports include negative interactions with emergency services. | AODFSV |
| Cohn, A. M., Zinzow, H. M., Resnick, H. S. & Kilpatrick, D. G. (2013) | USA | To examine  (a) the variance in reporting DAFR/IR and forcible rape (FR) to the police; (b) how incident characteristics might account for reasons victims did not report | Quantitative | 441 female rape victims who did not report to police (within a national telephone household probability sample) | DAFR/IR was positively and uniquely associated with ‘non-acknowledgment  of rape’ as a reason for not reporting the incident. DAFR/IR and FR were both positively and significantly associated with Criminal Justice Concerns. | Drug and alcohol-facilitated rape/Incapacitated rape (DAFR/ IR) |
| Crawford, E., O’Dougherty Wright, M. &   Birchmeier, Z. (2008) | USA | To explore university women’s perception of risk and behavioral changes when responding to risk in a DFSA scenario | Quantitative study of responses to vignettes | 406 female undergraduate students | Participants recognized some risk of having someone else pour their beer; but did not tend to perceive the risk of leaving one’s beer unattended. Little suspicion of presence of date-rape drug for those who fall ill at party. | DFSA |
| Du Mont, J., Macdonald, S. & Kosa, D. (2016) | Canada | To determine what factors are associated with cases classified as predatory DFSA | Quantitative prospective study | 184 cases of suspected intentional drugging from 35 hospital-based sexual assault treatment centers, collected June 2005 to March 2007 | Odds of having experienced DFSA were higher if the survivor self-reported mental health problems  in the previous 6 months or that the mode of suspected drugging was a recreational drug or non-alcoholic drink (versus an alcoholic drink) | DFSA |
| Du Mont, J., Macdonald, S., Rotbard, N., Asllani, E., Bainbridge, D. & Cohen, M. M. (2009) | Canada | To determine the prevalence of and factors associated with suspected drug-facilitated sexual assault. | Quantitative prospective study | 882 sexual assault victims from 7 of 35 hospital- based sexual assault treatment centers | 184 (20.9%) suspected of drug-facilitated sexual assault. Compared with other victims, these victims were more likely to have presented to a large urban center for care, to be employed, to have consumed  over-the-counter medications and street drugs in the 72 hours before being examined and to have used alcohol  before the assault. | DFSA |
| Dumbili, E. & Williams, C. (2020) | Nigeria | To explore how young Igbo ethnic group socially construct alcohol consumption and second to examine how the social construction of alcohol consumption facilitates the rape of female students | Qualitative research, thematic analysis | 31 male and female undergraduate students (aged 19-23 years) | Perspectives highlighted gendering of alcoholic beverages and men’s/women’s behaviors influenced by societal expectations. Gendering of alcoholic beverages facilitates date rape - offering women alcohol with this intent was disclosed by nearly all the men. | Alcohol facilitated sexual violence (AFSV) |
| Fields, L., Young, D. A., Patel, A. R., Munroe, C., Shumway, M, Bell, S. & Richer, L. A. (2022) | USA | To identify psychological impacts of DFSA and non-DFSA and determine how impaired trauma memory relates to development of PTSD and depression. | Retrospective case file analysis | 74 adults receiving sexual assault mental health services | Those who had voluntarily ingested substances had higher odds of a substance use disorder. Those who were administered substances without their knowledge experienced distress and self-blame. | DFSA |
| Finch, E. & Munro, V. E. (2007) | UK | To examine the extent to which the moral panics associated with ‘date-rape’ drugs and the social acceptability of alcohol influenced attributions of responsibility in mock jurors. | Qualitative, focus groups and mock trial simulations | Not reported | Participants showed questionable beliefs about level of intoxication of victim. Drug-assisted rape where victim was unaware of drugs was more unacceptable than victim intoxicated by own choice. | Date rape |
| French, B. H. & Nevile, H. A. (2012) | USA | To examine the relations between type of sexual coercion and health among Black and white students. | Quantitative survey | 221 female Black (*n* = 107) and white (*n* = 114) high school or university students, ranging from 14 to 19 years old. | Substance-facilitated coercion for Black participants indicated significantly lower self-esteem and increased psychological distress. | Substance facilitated sexual coercion |
| Gilbert, L., Sarvet, A. L., Wall, M., Walsh, K., Reardon, L., Wilson, P., Santelli, J., Khan, S., Thompson, M., Hirsch, J. S. & Mellins, C. A. (2019) | USA | To compare characteristics of incapacitated and non-incapacitated rape and to compare the situational contexts. | Quantitative, population-based survey of event-level data | 253 undergraduate women sexual assault victims reporting most significant sexual assault incident since entering college. | 47% of sample reported being incapacitated due to alcohol or drugs during the most significant incident.  Being at party before the event and “acquaintance” perpetrators were associated with incapacitated sexual assaults | Incapacitated rape (IR) |
| Gilmore, A. K., Walsh, K., Badour, C. L., Ruggiero, K. J., Kilpatrick, D. G. & Resnick, H. S. (2018) | USA | To examine the indirect association between rape tactics of forcible rape (FR) and drug or alcohol-facilitated/ incapacitated rape (DAFR/IR) | Quantitative | National samples of college (2,000) and household-residing (3,001) women. | 14.5% reported a lifetime history of FR and 7% DAFR/IR. Reporting of DAFR/IR in college sample (6.4%) compared to 7.4% in household-residing sample. Both FR and DAFR/IR were associated with recent suicidal ideation. | DAFR/IR |
| Girard, A. L. & Senn, C. Y. (2008) | Canada | To explore people’s attitudes and attributions when date rape drugs are involved in a rape. | Quantitative analysis of responses to date rape scenarios | 280 undergraduate students (Study 1: 143 male and 137 female; Study 2: 80 male and 80 female) | Perpetrators held more responsible and blameworthy, and victims blamed less where women were drugged deliberately or given alcohol without knowledge compared to when no drugs/alcohol involved. Women’s voluntary consumption of drugs prior to a sexual assault reduced perpetrator responsibility and blame and increased  blame to the victim compared to other situations (except in some cases, voluntary drunkenness). | Date rape |
| Gómez, P. T., Romo-Avilés, N. & Pavón-Benítez, P. (2021) | Spain | Investigate the factors associated with alcohol-facilitated sexual violence among young women in the Spanish night-time economy | Qualitative research | 26 Spanish adolescents (11 female, 15 male) aged between 16 – 22 years, recruited from educational centers. | Male participants described “opportunistic” forms of harassment/sexual abuse and the deliberate tactical use of alcohol and women’s inebriation. Women related specific protective behavioral strategies for drinking and sexual assault | AFSV |
| Jaffe, A. E., Blayney, J. A., Graupensperger, S., Cooper, R. & Larimer, M. E. (2022) | USA | To examine prepartying as both a risk factor and consequence of incapacitated rape (IR) among college women and men | Quantitative | 2,827 college women and men | Having a history of IR was associated with more alcohol consumption and blackouts when prepartying. Prepartying drinking was a prospective predictor of IR | IR |
| Jenkins, G. &   Schuller, R. A. (2007) | Canada | To explore the impact of complainant intoxication at the time of the alleged assault on jurors’ evaluation of DFSA case | Quantitative analysis of response to written trial stimulus | 171 (100 female, 71 male undergraduates) aged 18-51 years. | Negative forensic report without expert witness produced greater verdict leniency and more favorable evaluation of defendant’s case. No effect for complainant beverage consumption. Women more accepting of complainant account of assault as opposed to defendants (converse true for men). | DFSA |
| Krebs, C. P., Lindquist, C. H., Warner, T. D., Fisher, B. S. & Martin, S. L. (2009) | USA | To further understand the prevalence of campus sexual assault and the different types (completed, attempted, sexual battery) and how the assault occurred (ie through physical force, threatened force or incapacitation including DAFSV) | Quantitative, cross-sectional sexual assault victimization survey | 5,466 undergraduate women from 2 large public universities | Almost 20% experienced some type of completed sexual assault since entering college. Most sexual assaults occurred  after women voluntarily consumed alcohol. Few occurred after women had been given a drug without their knowledge or consent. | Alcohol-or other drug-enabled and DFSA |
| Larsen, M. &   Hilden, M. (2016) | Denmark | To provide descriptive data regarding male victims of sexual assault seen at the Centre for Victims of Sexual Assault in Copenhagen | Quantitative descriptive study | 55 male victims attending sexual assault center between March 2001 until December 2010; seen within 72 hours of the assault. | 29% (*n*=16) suspected they were victim of drug rape. Compared to female victims, male victims were more often assaulted by a stranger; more likely more than one perpetrator; more likely being victim of drug rape; less likely to have experienced previous sexual abuse and less willing to report to police | Drug-rape |
| Lawyer, S., Resnick, H., Bakanic, V., Burkett, T. & Kilpatrick, D. (2010) | USA | To examine the prevalence of drug-related sexual assaults, identify the frequency of assaults that occur following voluntary versus involuntary drug or alcohol consumption, and identify contextual correlates of drug-related assaults | Quantitative | 314 female undergraduate students | Drug-related sexual assaults occur more frequently than do forcible sexual assaults in a college-student population | DFR |
| Littleton, H., McConnell, A., Messman, T. L. & Layh, M. (2021) | USA | To identify types of rape using multiple assault characteristics, including survivor resistance and substance use by both survivor and perpetrator. | Quantitative survey | 344 cisgender undergraduate women who had experienced rape since the age of 14. | Women’s experiences of rape were shown to fall into five distinct typologies which were based on the presence of force or resistance and whether the victim and perpetrator were using substances. | Substance facilitated rape |
| McCauley, J. L., Conoscenti, L. M., Ruggiero, K. J., Resnick, H. S., Saunders, B. E. & Kilpatrick, D. G. (2009) | USA | Report on the prevalence, case characteristics and associated health risks of IS/DAFS using a large, nationally representative sample | Quantitative | 1,763 adolescent girls from 1995 aged between 12 and 17 years | 11.8% of adolescent girls reported some form of sexual assault. The prevalence of IS/DAFS was 2.1% of adolescent girls | DAFSA/ IR |
| McCauley, J. L., Ruggiero, K. J., Resnick, H. S. & Kilpatrick, D. G. (2010) | USA | To examine the relationship between rape and substance use problem as a function of forcible, incapacitated and DA rape | Quantitative | 1,998 US women aged 18-34 years | Lifetime experience of incapacitated rape was associated with increased odds of past-year binge drinking, marijuana use, and illicit drug use. Lifetime history of forcible rape and drug/alcohol facilitated rape were associated with increased odds of marijuana and illicit drug use | DAFR |
| McCauley, J. L., Ruggiero, K. J., Resnick, H. S., Conoscenti, L. M. & Kilpatrick, D. G. (2009) | USA | To examine the relationship between rape and substance use problems in college women as a function of forcible, incapacitated and substance-facilitated rape. | Quantitative | 1,980 college women aged 18-34 years | Lifetime prevalence of any type of rape was 11.3%. Prevalence estimates for binge drinking and substance abuse were 15.8% and 19.8%, respectively. Lifetime experience of IR and DFR but not FR associated with increased odds of past-year binge drinking and substance abuse | DFR/IR |
| Mgolozeli, S. E. & Duma, S. E. (2019) | South Africa | To determine and describe the types of rape experienced by men | Qualitative interpretive phenomenological analysis (IPA) | 11 adult males aged between 18 and 65 years who had experienced “fresh” rape victimization (less than 1 week old) presenting at a healthcare facility | Drug-facilitated rape was identified as a sub-type of gang rape where alcohol and drugs were used to intoxicate victims and facilitate rape. | DFR |
| Mognetti, B., Bo, M., Berta, G. N., Canavese, A., Castagna, P., Collini, F.m Santa, V., Salomone, A. & Gino, S. (2022) | Italy | To assess the characteristics of sexual violence where alcohol or other drugs were consumed among women attending a hospital. | Retrospective cross-sectional study of case files | 222 female patients, including 25 minors and 141 adults attending a local hospital. | 155 patients reported substance intake and 73% of those who drank alcohol had some amnesia, with 65% for those who took other substances. Those who took alcohol reported significantly higher risk of physical injury. | Alcohol and drug intake |
| Mokma, T. R., Esherlman, L. R. && Messman-Moore, T. L. (2016) | USA | To assess whether child, adult sexual abuse, self-blame, PTSD and alcohol use confer risk for specific types of adult sexual assault. | Quantitative survey | 929 female college students | Child sexual abuse indirectly predicted greater risk for substance-facilitated adult sexual assault. Broader results indicate the importance of assessing different risks for adult sexual assault. | Substance facilitated sexual assault |
| Moore, S. E. H. (2009) | UK | To explore the cultural construction of DFSA in British media | Media analysis Qualitative content analysis | Content analysis of news coverage in British media; questionnaires (200 undergraduate students) and interviews/focus groups (43 students) | DFSA is presented and received as a legitimate, plausible, and credible threat. The threat is frequently represented as both external and internal to the individual, resulting from an opportunistic attacker and/or one’s own negligent behavior. | DFSA |
| Moore, S. E. H. (2011) | USA | To look at the changing meaning of date rape in US newspapers | Media analysis | US newspapers over a fourteen-year period | “Date rape” has become a niche crime category and newsworthy over time. Argues that “date rape” has been recast to reinforce the reigning ideology of patriarchal society and deny the possibility that rape is a social and political problem. | Date rape |
| Prego-Meleiro, P., Montalvo, G., Garcí-Ruiz, C., Ortega-Ojeda, F., Ruiz-Pérez, I. & Sordo, L. (2021) | Spain | To assess the attitudes and perceptions of Spanish youth towards sexual violence within the nightlife context | Quantitative | 2,355 Spanish residents aged 18 – 24 years (gathered through snowball sampling from initial sample of university students) | Men were more willing to have intercourse with someone who could not consent due to drugs. Men saw women’s use of drugs, especially alcohol as the cause of/justifies sexual violence against women. Participants believed misconceptions around DFSA. Alcohol was underestimated. | DFSA |
| Quintana, J. M., García-Maroto, Á, Moreno, O. & Manzanero, A. L. (2020) | Spain | To analyze the cases of drug-facilitated sexual assault “chemical submission” from 2008 to 2017 that were registered in the area of the Spanish Civil Guard | Case analysis | 240 reported cases of aggression or sexual crimes facilitated by administration of drugs or medication | Upward trend of chemical submission cases over the years. Typical profile of victim is female, 25 years of Spanish nationality. Typical profile of perpetrator is male, 34 years of Spanish nationality, history of other crimes and no prior relationship with victim. | DFSA |
| Richer, L. A., Fields, L., Bell, S., Heppner, J., Dodge, J., Boccellari, A. & Shumway, M. (2017) | USA | To address methodological issues of nomenclature and drug-facilitated sexual assault (DFSA) operational definitions to improve case identification | Case analysis of health records | 390 patients suspected of drug-facilitated rape who presented acutely to rape center over 2-year period | Involuntary DFSA increased from 25% to 33% of cases over the 2-year period. No significant differences in severity of injury. DFSA victims presented sooner, and more often attended medical follow-up and psychotherapy than non-DFSA victims. | DFSA |
| Schramm, A. T., Swan, S. C., Lambdin, M. N., Fisher, B. S., Coker, A. L. & Williams, C. M. (2018) | USA | To examine the prevalence and risk of drugging in sexual minority university students | Quantitative | 5,691 university students | Male sexual minority college students  were 72.9% more likely to report drugging victimization than heterosexual males. No significant  differences were found for women. Both genders had greater drugging victimization among students who engaged in illicit drug use, binge drinking, and Greek life membership | Drugging |
| Singh, S. B., Meera, T. & Singh, T. B. (2014) | India | To identify the pattern of date rape cases in Imphal | Retrospective descriptive case analysis | 210 cases of sexual assault | 17 (8%) of cases were date rape victims; majority aged <20 years; over half assaults occurred in restaurants and in the afternoon. Consumption of alcohol offered by the perpetrator was present in 52% of cases. | DFSA |
| Stewart, D. N. & Jacquin, K. M. (2010) | USA | To examine the impact of a rape complainants’ willingness to ingest a chemical substance and the type of ingested substance on the decisions of mock jurors | Quantitative | 229 students recruited as mock jurors (66.8% female; 33.2% male), responding to 6 case summaries of fictional rape trial. | Jurors were influenced by the type of substance used by complainants, the willingness to use substances and rape myths. Complainants who willingly ingested a substance were viewed as less credible and more to blame than someone unwillingly ingested. | Ingestion of chemical substance |
| Swan, S. C., Woodbrown, V. D., Schramm, A. T., Warren, P. R., Lasky, N. V., Fisher, B. S., Bonsu, J. E., Coker, A. L. & Williams, C. M. (2017) | USA | To explore people’s motives for drugging, what drugs are used, how often it involves sexual assault, what are the outcomes for victims, are there patterns by gender and where does it occur | Quantitative | 6,064 students from 3 universities | More than 1 in 13 (7.8%) of students reported being drugged, and 83 students (1.4%) reported drugging someone. Perceptions of motivation were gendered, as were outcomes, with female victims suffering more negative consequences. | Drugging |
| Ullman, S. E., O’Callaghan, E. & Lorenz, K. (2019) | USA | To explore survivor perceptions of the different categories of drinking during AODFSV in order to develop trauma-informed practices. | Qualitative survey | 141 qualitative written responses from women who were drinking and/or using substances at the time of an assault. | The categories of AODFSV unimpaired (perceived no effect of drinking), impaired (conscious, but impacted by substance use), or incapacitated (unconscious due to substance use) only fit some experiences. Most responses were both impaired and incapacitated. | Alcohol/substance facilitated sexual assault |
| Walsh, K., DiLillo, D., Lanecky, A. & McChargue, D. (2013) | USA | To examine the potential risk factors for IR/DAFR (incapacitated/drug-or-alcohol facilitated rape) that may be unique from those associated with FR | Quantitative | 714 undergraduate women recruited from a large university (mean age 19.7 years) | History of child or adolescent sexual abuse (CASA) was positively associated with IR/DAFR and forcible rape (FR). Hyperarousal was the sole mechanism through which CASA was related to both IR/DAFR and FR. | DAFR/IR |
| Walsh, K., Zinzow, H. M., Badour, C. L., Ruggiero, K. J., Kilpatrick, D. G. & Resnick, H. S. (2016) | USA | To examine explanatory mechanisms in the pathway for rape types (FR vs DAFR/IR) to disparities in post-rape service seeking | Quantitative | 445 adult women from 2006 nationally representative household probability sample who had experienced FR, DAFR/IR, or both since age 14. | DAFR/IR-only victims were less likely to seek services compared with FR victims. FR victims are more likely to report fear and acknowledge incident as rape compared to DAFR/IR victims. | DAFR/IR |
| Wolitzjy-Taylor, K. B., Ruggiero, K. J., Danielson, C. K., Resnick, H. S., Hanson, R. F., Smith, D. W., Saunders, B. E. & Kilpatrick, D. G. (2008) | USA | To examine the lifetime prevalence of serious forms of dating violence in 12–17-year-olds, risk and protective factors and relation between dating violence and mental health | Quantitative | 3,614 youths (aged 12- to 17-year-olds) from 2005 nationally representative survey | Prevalence of dating violence was 1.6% (2.7% girls, 0.6% boys). 0.1% of sample reported experiencing drug/alcohol-facilitated rape (DAFR). Dating violence was associated with PTSD and major depressive episode. | DAFR |
| Zinzow, H. M., Resnick, H. S., McCauley, J. L., Amstadter, A. B., Ruggiero, K. J. & Kilpatrick, D. G. (2010) | USA | Examine the effects of lifetime exposure to FR, IR and DAFR tactics on risk for PTSD and depression | Quantitative | 2,000 college women national sample | 4.2% (*n*=84) reported history of IR, 2.7% reported history of DAFR (*n*=54), and 8.7% reported history of FR (*n*=174). All three rape tactics (FR, IR and DAFR) were associated with increased risk for PTSD and depression. | DAFR |
| Zinzow, H. M.  Resnick, H. S.,  Amstadter, A. B., McCauley, J. L., Ruggiero, K. J., Kilpatrick, D. G. (2010) | USA | To examine correlates of PTSD and depression in a community sample of women, with particular emphasis on evaluating the unique effects of lifetime exposure to three specific rape tactics: DAFR, FR, IR | Quantitative | 3,001 women aged 18-86 years) from nationally representative sample of English- or Spanish-speaking women | Three percent of women reported a history of IR (*n*=91), 2% reported history of DAFR (*n*=69), and 15% reported history of FR (*n*=439). History of DAFR and FR tactics were associated with PTSD. History of FR was associated with depression. | DAFR |
| Zinzow, H. M., Resnick, H. S., McCauley, J. L., Amstadter, A. B., Ruggiero, K. J. & Kilpatrick, D. G. (2012) | USA | To estimate prevalence of common psychiatric disorders with particular emphasis on distinguishing between DAFR, FR, IR | Quantitative | 3,001 women aged 18-86 years) from nationally representative sample of English- or Spanish-speaking women | History of DAFR and FR tactics associated with PTSD, major depressive episode and alcohol abuse. DAFR experiences associated with higher PTSD than FR. | DAFR |
